# Protoplasmic Astrocytes Enhance the Ability of Neural Stem Cells to Differentiate into Neurons *In Vitro*

**DOI:** 10.1371/journal.pone.0038243

**Published:** 2012-05-31

**Authors:** Yuan Liu, Li Wang, Zaiyun Long, Lin Zeng, Yamin Wu

**Affiliations:** The 3rd Department of Research Institute of Surgery, Daping Hospital, The Third Military Medical University, State Key Laboratory of Trauma, Burns and Combined Injury, Chongqing, People's Republic of China; Universitätsklinikum Carl Gustav Carus an der Technischen Universität Dresden, Germany

## Abstract

Protoplasmic astrocytes have been reported to exhibit neuroprotective effects on neurons, but there has been no direct evidence for a functional relationship between protoplasmic astrocytes and neural stem cells (NSCs). In this study, we examined neuronal differentiation of NSCs induced by protoplasmic astrocytes in a co-culture model. Protoplasmic astrocytes were isolated from new-born and NSCs from the E13-15 cortex of rats respectively. The differentiated cells labeled with neuron-specific marker β-tubulin III, were dramatically increased at 7 days in the co-culture condition. Blocking the effects of brain-derived neurotrophic factor (BDNF) with an anti-BDNF antibody reduced the number of neurons differentiated from NSCs when co-cultured with protoplasmic astrocytes. In fact, the content of BDNF in the supernatant obtained from protoplasmic astrocytes and NSCs co-culture media was significantly greater than that from control media conditions. These results indicate that protoplasmic astrocytes promote neuronal differentiation of NSCs, which is driven, at least in part, by BDNF.

## Introduction

The discovery that neural stem cells (NSCs) have the ability to regenerate functional neural cells has revolutionized neural tissue engineering and brought hopes to patients with central nervous system (CNS) disorders [1]. *In vitro*, NSCs can be expanded in the presence of mitogenic growth factors. Importantly, NSCs also have the potential to differentiate into neurons, astrocytes and oligodendrocytes in certain culture conditions [2].

Previous studies have suggested that the behavior of NSCs was regulated by both intrinsic characteristics and extrinsic signals originating from the culture medium. For example, epidermal growth factor (EGF) and basic fibroblast growth factor (bFGF) have been shown to promote the proliferation of NSCs in culture [3], [4]. In addition, nerve growth factors such as neurotrophin-3 (NT-3) [5] and ciliary neurotrophic factor [6] have also been reported to be key factors in regulating the differentiation of NSCs. Alternatively, signals from cell-cell contact can also regulate the multi-potential of NSCs. For example, amniotic stem cells can promote dendrites outgrowth of differentiated neurons arising from NSCs [7].

It has been well identified that astroyctes show neuroprotective effects, which enhances the survival of rat embryonic cortical and hippocampal neurons *in vitro* and *in vivo* [8], [9], [10]. But, there are two types of astrocytes in the CNS. For example, fibrous astrocytes are present in white matter, whereas protoplasmic astrocytes are located in grey matter [11], [12]. Furthermore, It was reported that mesencephalic protoplasmic astrocytes participated in the survival, maturation and neurogenesis of dopaminergic neurons through secreting neurotrophic factors, which indicated that protoplasmic astrocytes could induce proliferation and differentiation of NSCs during CNS development [13], [14], [15]. However, the functional interaction between NSCs and protoplasmic astrocytes remains unclear. In this study, we evaluated whether protoplasmic astrocytes are capable of promoting and supporting NSC differentiation into neurons *in vitro*. To examine a possible functional interaction, NSCs were divided into four groups: NSCs and protoplasmic astrocytes co-culture group, NSC and protoplasmic astrocytes co-culture+mouse anti-rat BDNF antibody group, NSCs cultured in NB+B27 medium group and NSCs cultured in DMEM/F12+10%FBS medium group.

## Methods

### 1. Culture of neural stem cells and incorporation of Brd-U

All experiments were approved by the Institutional Animal Care and Use Committee of the Third Medical Military University. Primary culture of NSCs was prepared from embryos 13.5 days of Wistar rats as described previously [16]. Briefly, the telencephalon was rapidly dissected and placed into 2 ml tubes containing 0.25% trypsin. The tissue was then mechanically dissociated into single cell suspension. Cell number and viability were assessed by staining a small volume of single cell suspension with 0.4% trypan blue. Single-cell suspension was then transferred to growth medium consisting of neurobasal medium which is suitable for the growth of fetal neural cells (NB, Hyclone, USA)) +2% B27(Gibico, USA) supplemented with 20 ng/ml human recombinant basic fibroblast growth factor (bFGF;Invitrogen Corporation) and 20 ng/ml epidermal growth factor (EGF; Invitrogen corporation,USA) at 4×10^5^ cells/ml. The cells were then plated into culture flasks and maintained at 37°C under a humidified atmosphere containing 5% CO2. After 5–6 d *in vitro*, the neurospheres were dissociated into single-cell suspension, diluted and seeded onto 96-well plates with 1–2 cells per well. The neurosphere subcultures were digested and another passage was performed as before. The cell passage protocol was performed every 6 days to obtained neurospheres originating from a single primary cell. Secondary or tertiary neurospheres were used for subsequent experiments.

For Brd-U incorporation, NSCs were incubated in growth medium containing 10 µM Brd-U for 18 hours prior to immunocytochemical analysis. For experiments involving differentiated NSCs, neurospheres were transferred into differentiating medium (DMEM/F12 containing 10% fetal bovine serum (Thermo, UT, USA) for 7 days.

### 2. Identification of NSCs

To examine the self-renewal and the potential ability to differentiate into neural lineage cells, neurospheres were stained with mouse anti-rat Brd-U (1∶800; Sigma-Aldrich, MO, USA) and mouse anti-rat Nestin (1∶600; Chemicon, MA, USA) antibodies respectively. Brd-U antibody labels dividing cells, while Nestin is a specific marker for neuroepithelial tissue. Cells positive for Brd-U staining indicates the ability to proliferate and self-renew. The differentiated cells from NSCs were characterized by antibodies, including rabbit anti-rat glial fibrillary acidic protein (GFAP; 1∶400; Sigma-Aldrich) which is a specific astrocyte marker, and the specific neuronal cell marker: mouse anti-rat β-tubulin III antibodies (1∶800; Sigma-Aldrich).Typical immunocytochemical procedures were used for identification the differentiated cells [17]. Briefly, the cells were fixed with 4% paraformaldehyde in PBS, permeabilized with 0.3% Triton X-100, and blocked with 5% normal goat serum. The cells were incubated with primary antibodies overnight at 4°C subsequently, then incubated with tetramethylrhodamine isothiocyanate (TRITC)-conjugated goat anti-rabbit and fluorescein isothiocyanate (FITC)-conjugated goat anti-mouse secondary antibodies (1∶100; Chemicon, MA, USA) for 1 h at 37°C and counterstained with 300 nM of 4–6-diamidino-2-phenylindole dihydrochloride (DAPI; Sigma-Aldrich) for 3 minutes. Labeled cells were scanned with a Leica confocal microscope (SP-2, Leica, Germany). For negative controls, the cells were processed by the same immunofluorescent staining technique, but with omission or pre-absorbtion of the primary antibodies.

### 3. Culture and identification of protoplasmic astrocytes

Protoplasmic astrocytes were prepared according to Karen Rosewater [12] with minor modification. Cortical tissue was collected from new-born 1–3 days rats and digested with 0.25% trypsin for 10 minutes, and then dissociated mechanically to single cell suspension. The cells were seeded at a density of 5×105/cm^2^ on poly-L-lysine treated 75 cm^2^ (Nunc, Roskilde, Danmark) with DMEM/F12 plus 10% fetal bovine serum (Thermo,USA). After 14 days of culture, 10 mmol/L L-leucine-methyl-ester (Sigma-Aldrich, USA) was added into the medium for 1 hour to kill microglia. Then the cells were quaked at a rate of 180 r/min for 16 hours to remove O-2A progenitors. The protoplasmic astrocytes were left in the flask and plated at an initial density of 5×10^5^/cm^2^. After 2 weeks of expansion, protoplasmic astrocytes were re-plated onto poly-L-lysine-treated 35 mm petri dishes at a density of 1×10^5^ cells/cm^2^ ml for co-culture. Immunostaining for GFAP was adopted to identify protoplasmic astrocytes.

### 4. Co-culture of NSCs and protoplasmic astrocytes

For co-culture of protoplasmic astrocytes and NSCs. Transwell dishes were used to produce a cell co-culture environment. NSCs were plated in poly-L-lysine pretreated six-well dishes (Nunc) and protoplasmic astrocytes were cultured in the insert (Millipore, MA, USA). The pore size of the membrane was 0.4 µm. Co-cultured cells were maintained in NB+2% B27 or NB+2%B27+BDNF antibody media. The concentration of BDNF antibody was 1 ug/ml. Half of the media were changed every 3 days. After 7 days of co-culture, the cells obtained from differentiated NSCs were fixed and labeled with neuronal specific marker β-tubulin III (1∶800; Sigma-Aldrich) and astrocyte-specific marker GFAP (1∶500; Sigma-Aldrich).

### 5. Western blot analysis

The differentiated cells were lysed and centrifuged at 14,000×g for 20 minutes. Protein concentration of the supernatant from the cell extract was determined using a BCA protein assay kit (Pierce Biotechnology, IL, USA).Equivalent amount of protein samples were loaded on SDS- polyacrylamide gels. After electrophoresis, the proteins were transferred to PVDF membranes and the blots were subsequently incubated with β-tubulin III or β-actin (1∶1000; Sigma-Aldrich) antibody respectively. For detection, horseradish peroxidase-conjugated secondary antibodies (1∶5000) were used followed by enhanced chemiluminescence detection (Millipore). The data were normalized by running parallel Western blots using β-actin as an internal control. The optical density was quantified with the Image-Pro Plus 6.0 software. Separate experiments were conducted for three times.

### 6. Enzyme linked immunosorbent assay (ELISA)

BDNF secretion by protoplasmic astrocytes was measured by mouse anti-rat BDNF ELISA kit (Roche, Shanghai, China).The supernatant solution from the various media conditions (seen above for details) was used as a sample following 1, 3, and 7 days after co-culture. The optical density (OD) was measured at 450 nm in a standard micro-plate reader (BioRad, PA, USA).Three independent experiments were conducted.

### 7. Statistical analysis

The percentage of differentiated neurons cells was calculated as the number of β-tubulin III-positive cells divided by the total number of neural stem cells (DAPI-stained cells) at 5 random fields under a 40× objective for each group in five independent experiments. Data are presented as mean ± SD. Statistical analysis was performed using one-way analysis of variance (ANOVA) followed by Dunnett's multiple comparison test. P<0.05 was considered to be statistically significant.

## Results

### NSCs have the ability to proliferate and differentiate into both neurons and astrocytes

**Figure 1:**
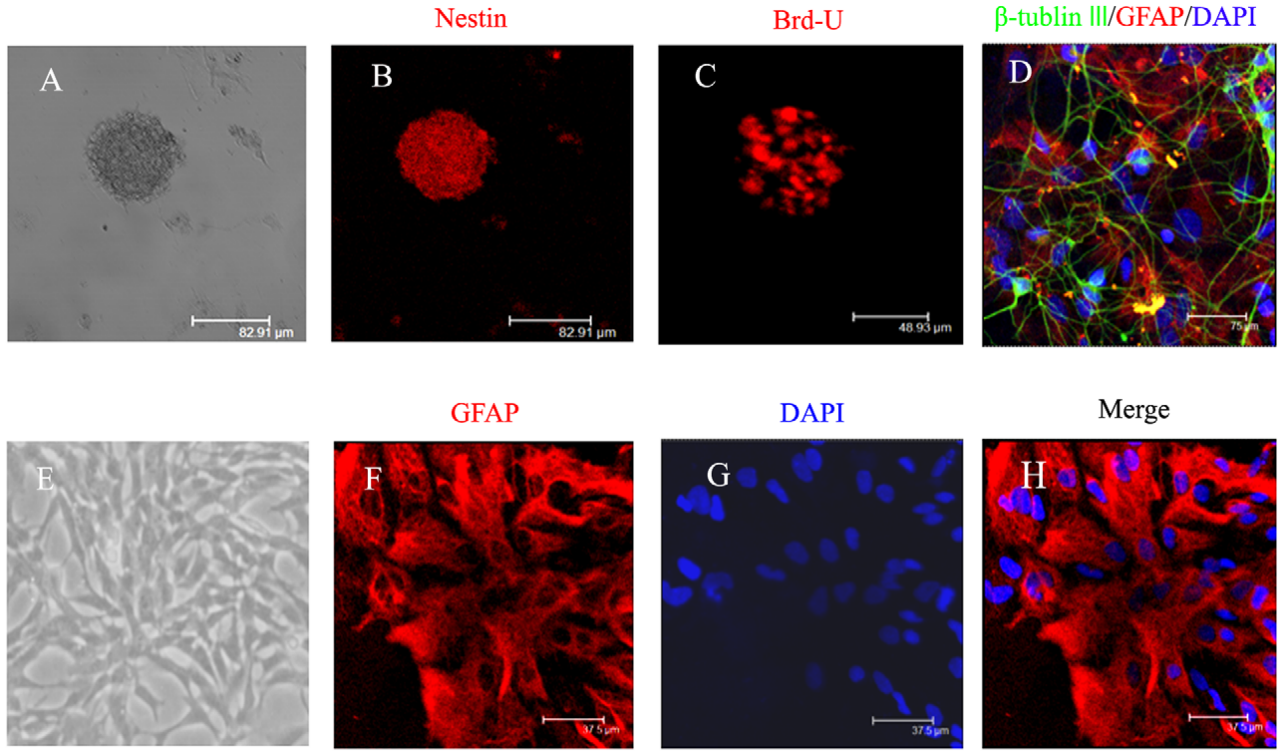
Identification of NSCs and protoplasmic astrocytes *in vitro*. (A) Representative photomicrograph of neurospheres in culture. (B) Immunocytochemical staining of purified NSCs with Nestin(bar = 82.91 µm). (C) Immunocytochemical staining of NSCs with anti-Brd-U antibody (bar = 48.93 µm). (D) Immunocytochemical staining of differentiated cells from NSCs (green indicates neuron-specific label β-tubulin; red indicates the astrocyte-specific label GFAP; blue indicates the nucleus-specific label DAPI; bar = 75 µm). (E) Representative photomicrograph of purified protoplasmic astrocytes (×100). (F) Immunocytochemical staining of purified protoplasmic astrocytes with GFAP. (G) Nucleus staining of purified protoplasmic astrocytes with DAPI. (H) Merge of F and G. The scale bar = 37.5 µm for F, G, and H.

On the second day of the primary culture, the cells growing as spheres in suspension are NSCs and express neural stem cell specific marker Nestin (Fig. 1A and B). Detection of DNA replication by Brd-U immunostaining of the spheres confirmed that these cells undergo proliferation (Fig. 1C). After withdraw of bFGF and EGF, the cells in spheres can differentiate into neurons and astrocytes as indicated with immunofluorescent staining by neuronal-specific marker β-tubulin III and astrocyte-specific marker GFAP, which indicated that the NSCs have a multi-differentiation potential. (Fig. 1D).

The purified protoplasmic astrocytes in the culture are flat and round, which have large cell bodies, wide cytodendrites and few cytodendritic branches. Additionally, immunofluorescent staining demonstrated that over 99% of the cells were positive for astrocyte specific marker GFAP (Fig. 1E–H).

### Effects of different culture conditions on NSC differentiation

**Figure 2:**
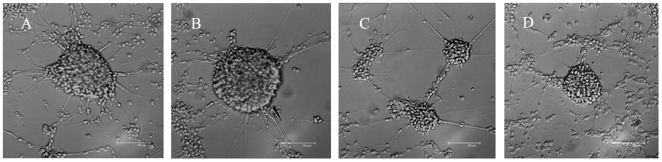
The growth and differentiation of NSCs with different media at 3 days *in vitro*. (A) Differentiation of NSCs co-cultured with protoplasmic astrocytes. (B) Differentiation of NSCs in NB+N2 medium. (C) Differentiation of NSCs in co-culture+BDNF antibody medium. (D) Differentiation of NSCs in DMEM/F12+10% FBS medium. The scale bar = 150 µm.

**Figure 3:**
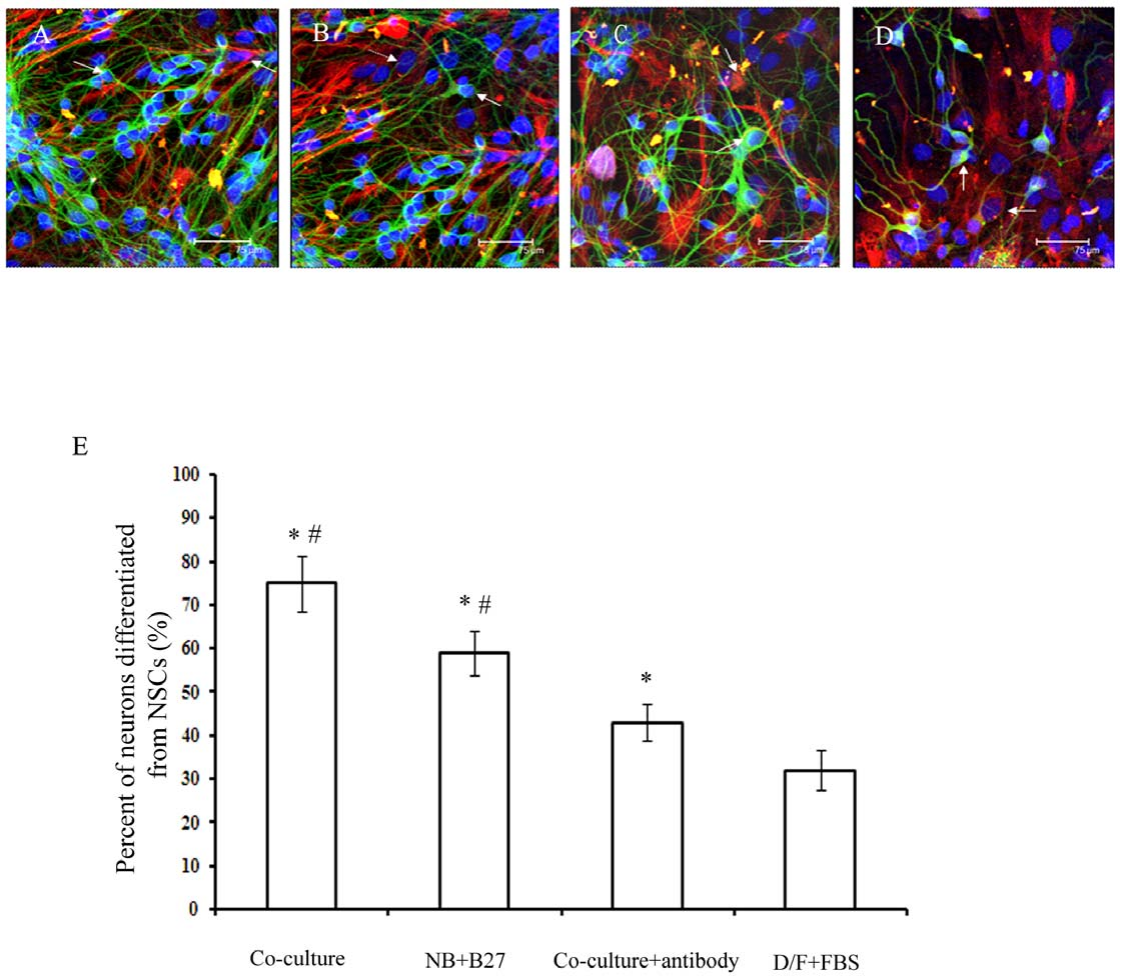
Quantification of differentiated cells cultured in various media at 7 days *in vitro*. Protoplasmic astrocytes grown in co-culture medium (A), NB+B27 medium (B), co-culture medium+BDNF antibody (C), and DMEM/F12+10% FBS medium (D). Scale bar = 75 µm. (E) Quantification of differentiated neurons in the various media conditions. β-tubulin III staining (green) indicates neurons; GFAP staining (red) indicates astrocytes. The nuclei were counterstained with DAPI (blue). *p<0.05 indicates statistical significance compared with the D/F+FBS group. ^#^ p<0.05 indicates statistical significance compared with the co-culture+antibody group.

On the second day after plating, the NSCs co-cultured with protoplasmic astrocytes began to adhere to the culture wall and grew out thin and short cytodendrites. After three days, the differentiated cells that migrated away from the spheres developed morphological characteristics of neurons, including spherical and refractile cell bodies with neurite-like processes (Fig. 2). Seven days after co-culture, a large proportion of cells exhibited thin and long cytodendrites, and the cytodendrites began to form connections with other cytodendritic processes. In fact, these cells were β-tubulin III immunoreactive positive neurons and were more abundant in protoplasmic astrocyte co-culture medium than those in the other three media (Fig. 3A–D and Fig. S1, Fig. S2, Fig. S3, Fig. S4).

To confirm differentiated cells derived from NSCs in the various culture conditions, specific markers for astrocytes and neurons were used for immunofluorescent staining. Our results demonstrated that 7 days after co-culturing, more differentiated cells in the co-culture medium expressed the neuronal specific marker β-tubulin III, (75%±6.4% vs. 59%±5.2% in the NB+B27 group, 43%±4.2% in the co-culture+BDNF antibody and 32%±4.6% in DMEM+10% FBS group; Fig. 3E), Which indicated that protoplasmic astrocytes and NSCs co-culture medium is more suitable for the neuronal differentiation of NSCs *in vitro*.

**Figure 4:**
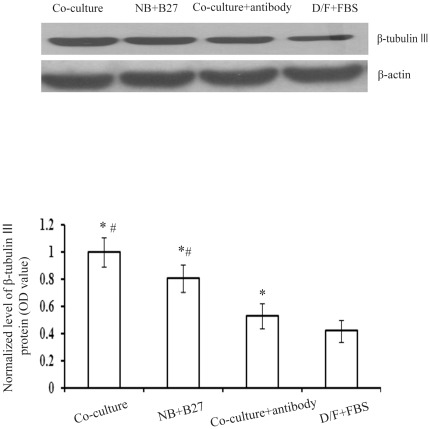
Western blot analysis of β-tubulin III protein in differentiated cells cultured with various media at 7 days *in vitro*. (A) Western blots showing the relative amount of β-tubulin **III** protein in all groups. β-actin was used to control for loading. (B) Data represent mean ± SD of three independent experiments. *p<0.05 indicates statistical significance compared with the D/F+FBS group. ^#^ p<0.05 indicates statistical significance compared with the co-culture+antibody group.

Moreover, western blot analysis confirmed β-tubulin III expression in differentiated neurons of the four various media conditions. After 7 days of differentiation *in vitro*, the expression level of β-tubulin III in the co-culture medium was greater than those in the other three media conditions. In fact, the quantity of β-tubulin III in co-culture+BDNF antibody was apparently less than that in co-culture medium. Therefore, blocking the effects of BDNF may inhibit neuronal differentiation of NSCs *in vitro* (Fig.4).

### BDNF mediates protoplasmic astrocyte-induced neuronal differentiation of NSCs

To identify the level of BDNF secretion from protoplasmic astrocytes, the content of BDNF in the different media conditions was examined by ELISA at different time points *in vitro*. One day after culture preparation, the concentration of BDNF in the protoplasmic astrocytes and NSCs co-culture medium condition was 67.2±5.3 pg/ml. Interestingly, BDNF dramatically increased to, 85.2±6.2 pg/ml on the 3rd day, and further increased to 97.1±7.4 pg/ml on the 7th day. The content of BDNF in NSCs cultured in NB+B27 media was higher than that in the DMEM/F12+FBS medium at each of the time points, but less than that in the NSCs co-cultured with protoplasmic astrocytes medium (Table 1).

**Table 1:** BDNF secretion in supernatant solution of different media.

	1 day	3 day	7 day
PAS and NSCs co- culture media	67.2±5.3	85.2±6.2	97.1±7.4
NB+B27 media	43.4±4.9*	50.7±5.1*	58.6±6.8*
DMEM/F12+FBS media	21.4±3.6*	27.9±4.1*	23.2±3.9*

Units = pg/ml for all data in the table.

N = 3 for all groups;

* indicates p<0.05 compared to the protoplasmic astrocyte group.

## Discussion

Cell transplantation is becoming an important strategy for the treatment of neurological disorders. Because NSCs have the potential of self-renewal and multi-lineage differentiation, transplantation of NSCs has been considered to be a promising therapy for disease and injuries of central nervous system. [18]. However, some studies have shown that proliferation of transplanted NSCs is limited [19] and the majority of NSCs differentiated into astrocytes, rather than neurons in the damaged lesions. Therefore, maintaining the self-renewal capacity of NSCs and controlling their directed differentiation into specific neural cell types is a major challenge for the treatment of central nervous system disease [20].

Less clear are the molecular mechanisms that regulate the differentiation of NSCs and how these might affect the neurogenesis of NSCs under pathological conditions. Recent studies have demonstrated that, in addition to the intrinsic properties of neural stem cells, the extrinsic local microenvironment or “niche”, including growth factors, cytokines and cell-cell contact, plays a key role in determining the fate of neural stem cells [19], [21], [22], [23].

Differentiation of NSCs *in vitro* has been examined using a variety of methods. For example, co-culturing NSCs with amniotic stem cells, adipose stromal cells and activated astrocytes has been performed by several groups. Some studies revealed that sequential treatment with NT-3 and bFGF significantly promoted the generation of more β-tubulin III positive cells, as well as, fewer GFAP positive cells from NSCs [7], [10], [24]. These results suggest that the microenvironment plays a prominent role for the induction of NSCs to differentiate into neurons.

In order to investigate the effects of the various culture media on NSCs differentiation *in vitro*, in this experiments, four types of culture conditions were used. Among which, NB+B27 medium was beneficial for the growth of neurons, while DMEM/F12+10% FBS medium was found to induce differentiation of NSCs into astrocytes. Remarkably, protoplasmic astrocytes co-culture medium provided sustaining neurotrophic support for NSCs. Co-culture+BDNF antibody medium, on the other hand, selectively blocked the effect of BDNF during NSCs differentiation. In the first three days of initial co-culture, NSCs in all groups began to adhere and differentiate into neuron-like cells. Seven days after co-culture, the cell originating from NSCs co-cultured with protoplasmic astrocytes were more likely to differentiate into neurons than those from NSCs in NB+B27,co-culture+BDNF antibody and DMEM+10%FBS media.Moreover, the level of β-tubulin III protein was consistent with the immunocytochemical staining results in all groups. In fact, we found that a few of NSCs maintained morphological characteristics typical of neurons and expressed β-tubulin III neuronal-specific marker following co-culture+BDNF antibody post-induction. These results suggest that protoplasmic astrocytes have an effect on the differentiation of NSCs at the level of lineage determination, which can promote NSCs differentiating into more neurons *in vitro*.

It has been identified that astrocytes can secreted neurotrophins (NGF, CNTF, BDNF, NT-3), cytokines (LIF), chemokines (IL-4,IL-6) and protein of transcript factors (p-state3) under certain conditions, through which it can affect the proliferation, differentiation and migration of NSCs. It was reported by Freda Miller that astrocytes can promote the dendrite formation of NSCs by secreted NGF through MEK-ERK signaling pathway. The research of Arturo Alvarez-Buylla indicated that robo2 protein secreted from astrocytes can promote the migration of NSCs in hippocampus through JAK-STAT pathway. At the same time others have identified that chemokines IL-6, IL-4 secreted from activated astrocytes stimulated by LPS or erythropoietin can inhibit the differentiation of NSC's to neurons [10], [25], [26].The effect of different phenotype of astrocytes is not the same. It has been reported that protoplasmic astrocytes produce a neuroprotective effect on the survival of neurons in the midbrain by secreting neurotrophic factors. Among these neurotrophic factors, BDNF is a particularly important positive regulator of neurogenesis. [12]. BDNF is a protein that belongs to the family of neurotrophin and regulates the survival, growth and migration of neurons in the CNS [27], [28]. Besides the effect of BDNF on the long-term modification of neuronal excitability and synaptic function, studies have demonstrated that BDNF enhanced the formation, differentiation of neurons derived from neural stem cells *in vitro* [29,29]. *In vivo*, BDNF delivery to SVZ progenitor cells increases neuronal recruitment to the lesion and results in ectopic addition of newly generated neurons, expressing markers of dopaminergic neurons, in the Alzheimer's disease adult rat brain [30], [31].

In the current study, we observed that under the protoplasmic astrocytes co-culture condition, the ratio of NSCs differentiating into neurons was enhanced relative to those in the NB+B27, the co-culture+BDNF antibody and the DMEM/F12+10% FBS media conditions. Moreover, to determine the role of neurotrophic factors in the different media conditions, we examined the level of BDNF at different time points following co-culture plating. The concentration of BDNF in the protoplasmic astrocytes and NSCs co-culture medium condition was significantly higher than those in the NB+B27 and the DMEM/F12+FBS media condition at different time points, which indicated that protoplasmic astrocytes can provide sustaining neurotrophic support for NSCs through secreting BDNF. Thus, it appears that BDNF secretion by protoplasmic astrocytes may play an important role in inducing the differentiation of NSCs into neurons. Importantly, owing to the short half-life of BDNF, the sustained secretion of BDNF from protoplasmic astrocytes may provide a novel means to support the differentiation of NSCs into neurons.

In summary, NSCs co-cultured with protoplasmic astrocytes may have a tendency to effectively differentiate into more neurons relative to the other culture conditions. This finding suggests that protoplasmic astrocytes co-cultured with NSCs could be especially useful for neural tissue engineering. Nonetheless, additional studies will be required to determine whether the differentiated neurons from NSCs are capable of surviving and migrating to appropriate location after implantation into the lesion site. Also important will be determine whether these NSCs-derived neurons are able to improve neurological functions under disease conditions. Finally, the signaling pathways that control neuronal differentiation in the co-culture conditions are still in need of future research.

## Supporting Information

Figure S1 **The immunocytochemical staining of differentiated cell from NSCs co-cultured with protoplasmic astrocyte.** **β-tubulin III staining (green) indicates neurons; GFAP staining (red) indicates astrocytes.** The nuclei were counterstained with DAPI (blue).
(JPG)

Figure S2 **The immunocytochemical staining of differentiated cell from NSCs grown in NB+B27 medium. β**-tubulin III staining (green) indicates neurons; GFAP staining (red) indicates astrocytes. The nuclei were counterstained with DAPI (blue).
(JPG)

Figure S3 **The immunocytochemical staining of differentiated cell from NSCs grown in co-culture+BDNF antibody medium.** **β-tubulin III staining (green) indicates neurons; GFAP staining (red) indicates astrocytes.** The nuclei were counterstained with DAPI (blue).
(JPG)

Figure S4 **The immunocytochemical staining of differentiated cell from NSCs grown in DMEM+10%FBS medium.** β-tubulin III staining (green) indicates neurons; GFAP staining (red) indicates astrocytes. The nuclei were counterstained with DAPI (blue).
(JPG)
