# Supplementary figures and images for: Protoplasmic Astrocytes Enhance the Ability of Neural Stem Cells to Differentiate into Neurons In Vitro
Source: PLoS One. 2012 May 31;7(5):e38243. doi: 10.1371/journal.pone.0038243 (PMC3365019; doi:10.1371/journal.pone.0038243)

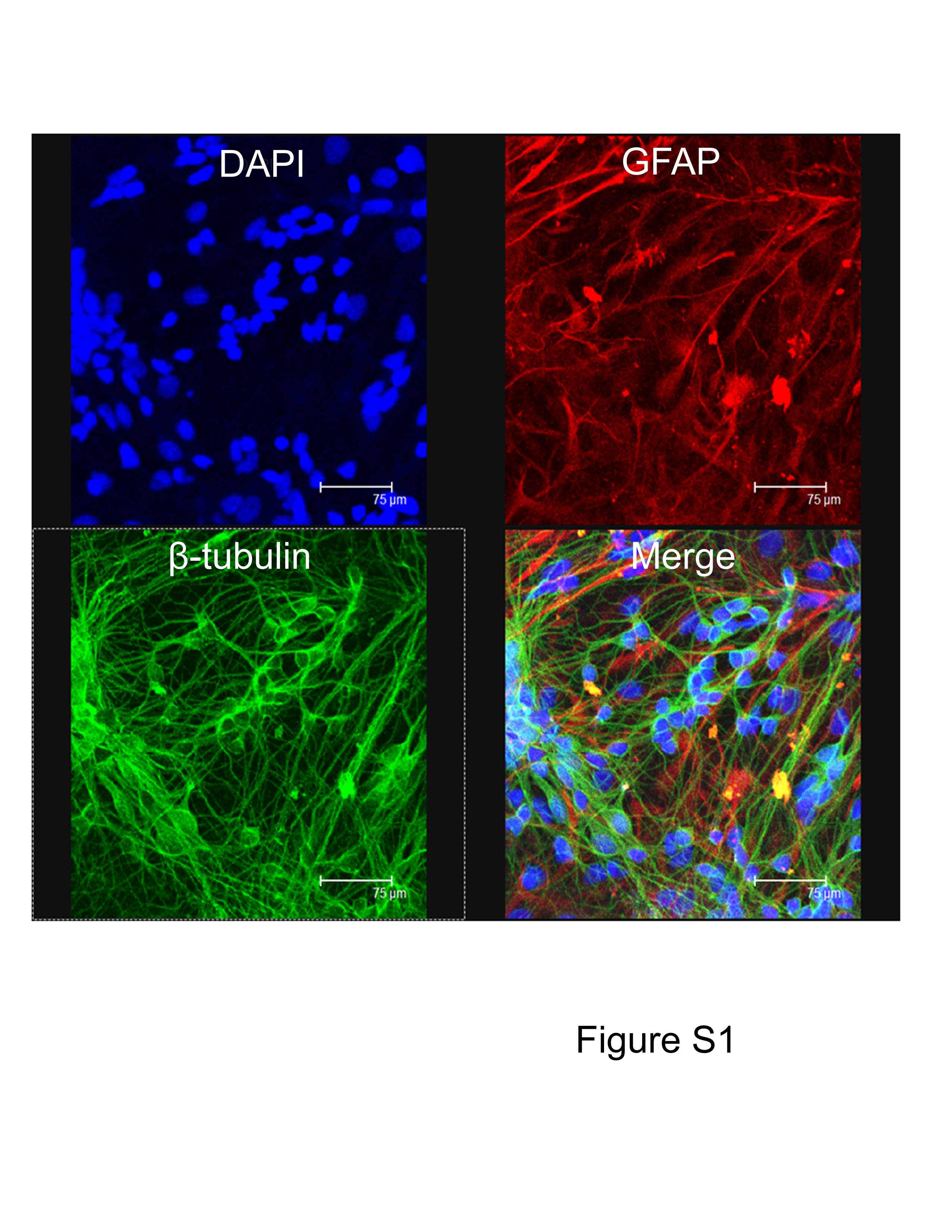

Supplement: Figure S1 — The immunocytochemical staining of differentiated cell from NSCs co-cultured with protoplasmic astrocyte. β-tubulin III staining (green) indicates neurons; GFAP staining (red) indicates astrocytes. The nuclei were counterstained with DAPI (blue). (JPG) [file pone.0038243.s001.jpg]

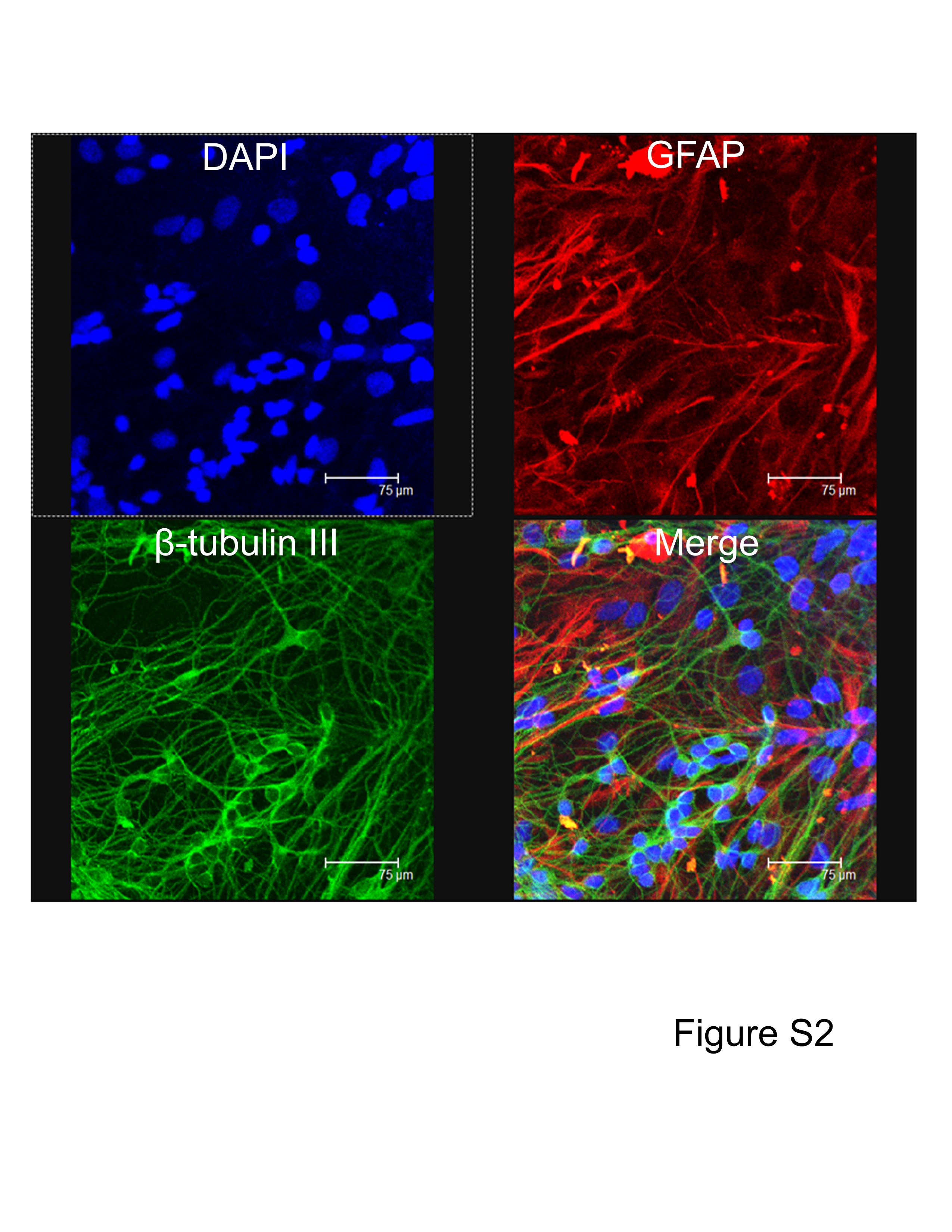

Supplement: Figure S2 — The immunocytochemical staining of differentiated cell from NSCs grown in NB+B27 medium. β-tubulin III staining (green) indicates neurons; GFAP staining (red) indicates astrocytes. The nuclei were counterstained with DAPI (blue). (JPG) [file pone.0038243.s002.jpg]

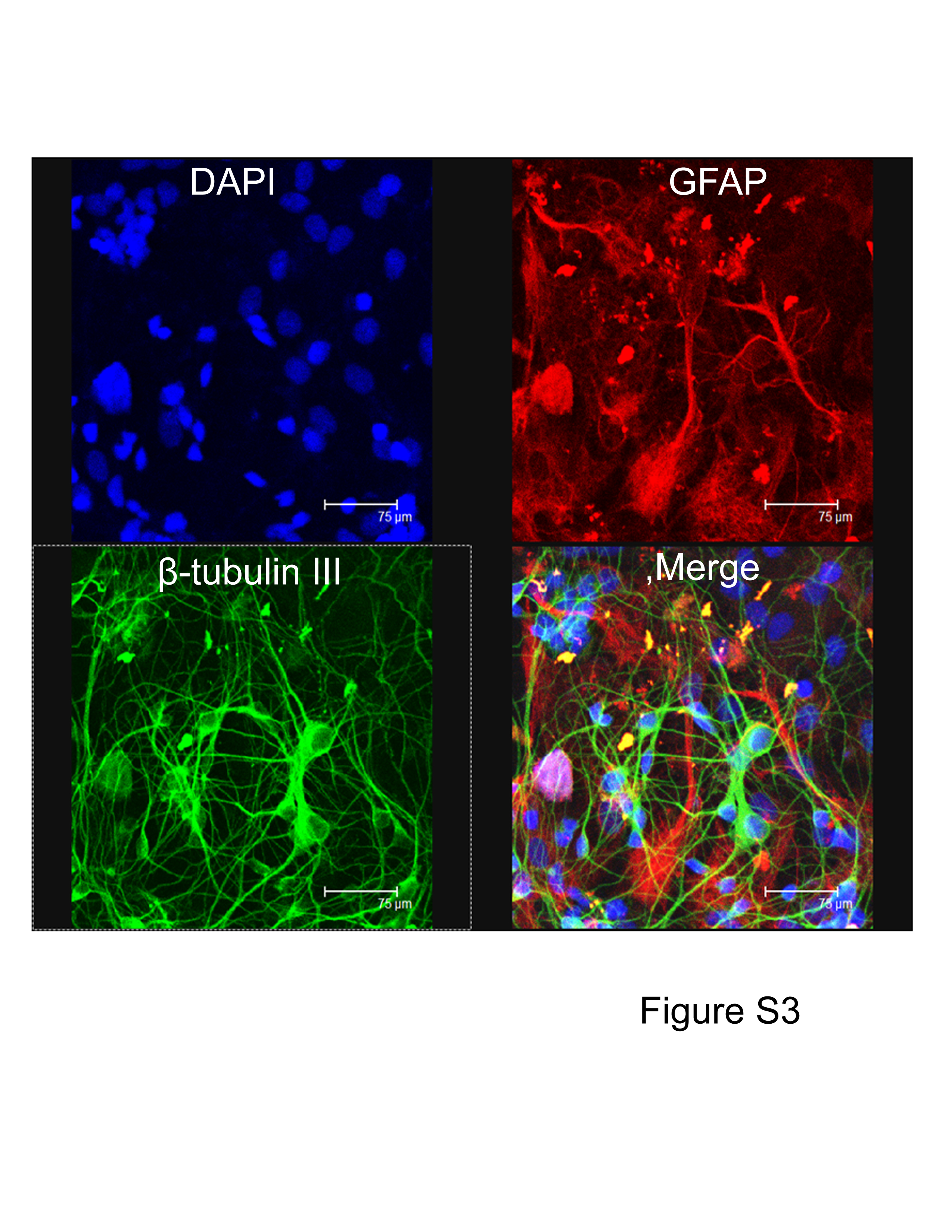

Supplement: Figure S3 — The immunocytochemical staining of differentiated cell from NSCs grown in co-culture+BDNF antibody medium. β-tubulin III staining (green) indicates neurons; GFAP staining (red) indicates astrocytes. The nuclei were counterstained with DAPI (blue). (JPG) [file pone.0038243.s003.jpg]

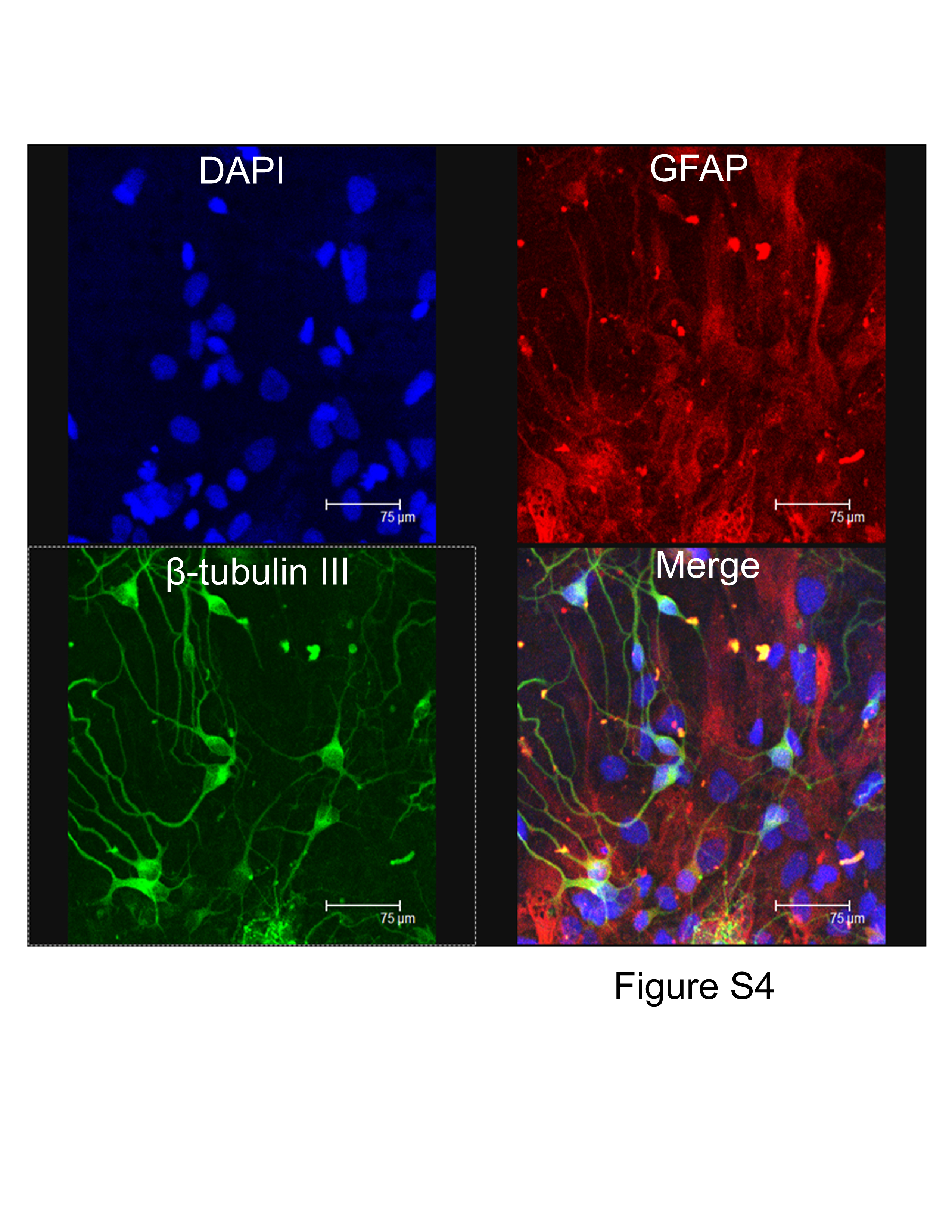

Supplement: Figure S4 — The immunocytochemical staining of differentiated cell from NSCs grown in DMEM+10%FBS medium. β-tubulin III staining (green) indicates neurons; GFAP staining (red) indicates astrocytes. The nuclei were counterstained with DAPI (blue). (JPG) [file pone.0038243.s004.jpg]
